# p62/sequestosome 1 in human colorectal carcinoma as a potent prognostic predictor associated with cell proliferation

**DOI:** 10.1002/cam4.1093

**Published:** 2017-05-23

**Authors:** Shun Nakayama, Hideaki Karasawa, Takashi Suzuki, Shinichi Yabuuchi, Kiyoshi Takagi, Takashi Aizawa, Yoshiaki Onodera, Yasuhiro Nakamura, Mika Watanabe, Fumiyoshi Fujishima, Hiroshi Yoshida, Takanori Morikawa, Tomohiko Sase, Takeshi Naitoh, Michiaki Unno, Hironobu Sasano

**Affiliations:** ^1^ Department of SurgeryTohoku University Graduate School of MedicineSendaiJapan; ^2^ Department of PathologyTohoku University HospitalSendaiJapan; ^3^ Department of Pathology and HistotechnologyTohoku University Graduate School of MedicineSendaiJapan; ^4^ Department of Anatomic PathologyTohoku University Graduate School of MedicineSendaiJapan

**Keywords:** Cell proliferation, colorectal carcinoma, immunohistochemistry, p62, prognosis

## Abstract

p62/sequestosome 1 (p62) is a multi‐domain protein that functions as a receptor for ubiquitinated targets in the selective autophagy and serves as a scaffold in various signaling cascades. p62 have been reported to be up‐regulated in several human malignancies, but the biological roles and significance of p62 are still poorly understood in colorectal carcinoma. We immunohistochemically evaluated p62 in 118 colorectal adenocarcinoma and 28 colorectal adenoma cases. We used four colon carcinoma cells (HCT8, HT29, COLO320, and SW480) in the in vitro studies. p62 immunoreactivity was detected in 11% of colorectal adenoma cases and 31% of adenocarcinoma cases, while it was negligible in the normal epithelium. The immunohistochemical p62 status was significantly associated with synchronous liver metastasis, and it turned out to be an independent adverse prognostic factor in colorectal cancer patients. Following in vitro studies revealed that HCT8 and HT29 cells transfected with p62‐specific siRNA showed significantly decreased cell proliferation activity, whereas COLO320 and SW480 cells transfected with p62 expression plasmid showed significantly increased cell proliferation activity. The p62‐mediated cell proliferation was not associated with the autophagy activity. These findings suggest that p62 promotes the cell proliferation mainly as a scaffold protein, and that the p62 status is a potent prognostic factor in colorectal carcinoma patients.

## Introduction

Colorectal carcinoma is the third most common newly diagnosed malignancy and the third leading cause of cancer‐related death in both men and women in the United States 1. The prognosis of colorectal cancer has been recently improved due to advances in chemotherapy and target‐specific therapies such as anti‐epidermal growth factor receptor (EGFR) antibody therapy 2. However, the estimated 5‐year overall survival for early‐stage colorectal carcinoma is 90%, decreasing to 71% for regional disease and 13% for distant disease, respectively 1. Therefore, it has becomes critical to find out clinical and biological markers that predict recurrence and also determine the postoperative algorithm of treatment in individual patients.

A multifunctional adapter protein, mammalian sequestosome 1 (p62/SQSTM1), hereinafter referred to as p62, was identified as an ubiquitin‐binding protein, transporting ubiquitinated proteins into autophagosomes through selective autophagy 3, 4, 5. In addition, p62 could activate several intracellular signaling pathways, such as nuclear factor‐kappa‐B (NF‐*κ*B), nuclear factor erythroid 2‐related factor 2 (NRF2), and mammalian target of rapamycin (mTOR) signalings, as a multifunctional scaffold protein 6, 7, 8. p62 is constantly degraded by autophagy ancillary to ubiquitinated proteins under physiological conditions 3, 4, 5. However, emerging evidence has revealed that p62 protein accumulates in several types of malignant tumors such as gastrointestinal, prostate, liver, breast, and lung carcinomas 9, 10, 11, 12, 13, 14, 15, 16, 17, and it has been reported that p62 accumulation is related to adverse clinical outcomes in the patients of lung cancer 9 and triple‐negative breast cancer 16. p62 immunolocalization has been reported in colorectal carcinomas 10, 14, 15, but its biological functions and clinical significance, including correlation with the clinical outcome of patients, remain largely unknown. In this study, we examined p62 immunoreactivity in human colorectal carcinoma and the biological functions of p62 in human colon carcinoma cell lines.

## Material and Methods

### Patients and tissues

A total of 118 colorectal carcinoma specimens were obtained from patients who underwent surgical treatment between 2000 and 2006 in the Department of Surgery at Tohoku University Hospital, Sendai, Japan. None of the patients studied received irradiation or chemotherapy prior to surgery in our study. Patients clinically suspected to have hereditary nonpolyposis colorectal cancer or carcinoma associated with inflammatory bowel disease were excluded from this study. Thirty‐eight patients with TNM stages II and III colorectal carcinoma received 5‐fluorouracil‐based adjuvant chemotherapy. The mean follow‐up time was 69.8 months (range, 2–131 months). In this study, we also used 28 colon adenoma specimens obtained from 28 patients who underwent surgery to extirpate colorectal carcinoma between 2007 and 2010 in the Department of Surgery at Tohoku University Hospital. All specimens had been fixed in 10% formalin and embedded in paraffin wax.

Informed consent was obtained from all the patients, and the Ethics Committees at Tohoku University School of Medicine approved the research protocols for this study (approval number: 2011‐597).

### Immunohistochemistry

Mouse monoclonal antibody for human p62 (clone 5F2) was purchased from Medical and Biological Laboratories Corporation (MBL; Nagoya, Japan). The characteristics of this antibody used for both immunohistochemistry and immunoblotting were previously described 15, 18. Mouse monoclonal antibody for Ki‐67 (clone MIB‐1) was purchased from DAKO (Carpinteria, CA). A Histofine Kit (Nichirei, Tokyo, Japan), using the streptavidin‐biotin amplification method, was used for immunohistochemistry in this study. Antigen retrieval for p62 was performed by heating the slides in a microwave oven for 15 min in citric acid buffer (2 mmol/L citric acid and 9 mmol/L trisodium citrate dehydrate, pH 6.0), and for Ki‐67, immunostaining was done by heating the slides in an autoclave at 120°C for 5 min in citric acid buffer. The dilutions of the primary antibodies used in this study were as follows: p62, 1:500; and Ki‐67, 1:100. The antigen‐antibody complex was visualized with 3,3′‐diaminobenzidine solution [1 mmol/L 3,3′‐diaminobenzidine, 50 mmol/L Tris‐HCl buffer (pH 7.6), and 0.006% H_2_O_2_] and counterstained with hematoxylin. As a negative control, normal mouse IgG (Negative Control Mouse IgG1, DAKO) was used instead of the primary antibodies, and no immunoreactivity was detected in these tissues.

p62 immunoreactivity was detected in the cytoplasm, and the cases that had more than 10% positive cells were considered positive, according to a previous report 9. Immunohistochemical staining for Ki‐67 was detected in the nucleus, and the percentage of immunopositive cells [i.e., labeling index (LI)] was determined.

### Cell lines and chemicals

Four human colon carcinoma cell lines (HCT8, HT29, COLO320, and SW480) and one normal colon epithelial cell line (CCD841) were provided from the American Type Culture Collection (Manassas, VA), and subjected to short tandem repeat (STR) genomic profiling for authentication by BEX Co. Ltd. (Tokyo, Japan) using the CELL ID System (Promega, Madison, WI). The cells were cultured at 37°C with 5% CO_2_ in RPMI 1640 Medium (Sigma‐Aldrich, St. Louis, MO), containing 10% heat‐inactivated fetal bovine serum (FBS; Sigma‐Aldrich) and 1% penicillin–streptomycin (Invitrogen, Carlsbad, CA). The cell blocks of these cell lines were prepared according to the fibrin clot method reported by Furtado 19 and embedded in paraffin wax. Autophagy inhibitor bafilomycin A1 was purchased from Sigma‐Aldrich (St. Louis, MO).

### Small interfering RNA transfection

Two small interfering RNAs (siRNAs) for p62 were purchased from Sigma‐Aldrich. The target sequences of siRNA against p62 were as follows: si1, 5′‐GGCUGAAGGAAGCUGCCUUd(TT)‐3′ (sense) and 5′‐AAGGCAGCUUCCUUCAGCCd(TT)‐3′ (anti‐sense); and si2, 5′‐CACUUCGGGUGGCCAGGAUd(TT)‐3′ (sense) and 5′‐AUCCUGGCCACCCGAAGUGd(TT)‐3′ (anti‐sense). MISSION siRNA Universal Negative Control (Sigma‐Aldrich) was also used as a negative control (siC). Cells were transfected with siRNA (final concentration, 10 nmol/L) using Lipofectamine RNAiMAX transfection reagent (Invitrogen).

### Plasmid transfection

Hemagglutinin (HA)‐tagged p62 plasmid (Addgene plasmid 28027) 20 and monomeric red fluorescence protein (mRFP)‐green fluorescent protein (GFP) tandem fluorescent‐tagged microtubule‐associated protein 1 light chain 3 (tfLC3) plasmid (Addgene plasmid 21074) 21 were purchased from Addgene (Cambridge, MA). The HA‐tagged p62 plasmid and control plasmid pcDNA4/TO (Invitrogen) were transfected into the cells using Lipofectamine LTX and PLUS reagent (Invitrogen), and the tfLC3 plasmid (final concentration, 1 ng/*μ*L) was transfected into the cells using Lipofectamine 2000 reagent (Invitrogen) with or without siRNAs (final concentration, 8 nmol/L) according to the manufacturer's instructions.

### Real‐time PCR

Total RNA of HCT8 and HT29 cells was extracted 2 days after siRNA transfection using TRIzol reagent (Invitrogen), and cDNA was synthesized using a QuantiTect reverse transcription kit (Qiagen, Venlo, The Netherlands). Real‐time PCR was carried out using the LightCycler System and FastStart DNA Master SYBR Green I (Roche Diagnostics, Mannheim, Germany). The PCR primer sequence used in this study was as follows. p62 (NM_003900): forward 5′‐ TCCTGCAGACCAAGAACTATGACATCG ‐3′ and reverse 5′‐ TCTACGCAAGCTTAACACAACTATGAGACA ‐3′, and ribosomal protein L13A (RPL13A) (NM_012423): forward 5′‐CCTGGAGGAGAAGAGGAAAGAGA‐3′ and reverse 5′‐TTGAGGACCTCTG‐ TGTATTTGTCAA‐3′. p62 mRNA levels were summarized as the ratio of RPL13A mRNA level (%) in this study.

### Immunoblotting

The protein of colon carcinoma cells was extracted using M‐PER Mammalian Protein Extraction Reagent (Pierce Biotechnology, Rockford, IL) with Halt Protease Inhibitor Cocktail (Pierce Biotechnology). Twenty micrograms of the protein were subjected to SDS‐PAGE (15% acrylamide gel). The primary antibodies used in this study were p62 used for immunohistochemistry (MBL), rabbit polyclonal antibodies for microtubule‐associated protein 1 light chain 3 (LC3; MBL), rabbit monoclonal antibodies for glyceraldehyde‐3‐phosphate dehydrogenase (GAPDH; Cell Signaling Technology, Danvers, MA), and mouse monoclonal antibody for *β*‐actin (clone AC‐15; Sigma–Aldrich) (1:1000 dilution, respectively). Antibody‐protein complexes on the blots were detected using ECL Plus western blotting detection reagents (GE Healthcare, Buckinghamshire, UK), and the protein bands were visualized with LAS‐1000 image analyzer (Fuji Photo Film Co., Tokyo, Japan).

### Cell proliferation assay

HCT8 (3000 cells/well), HT29 (5000 cells/well), COLO320 (10,000 cells/well), and SW480 (10,000 cells/well) cells were seeded in 96‐well plates, and incubated in RPMI‐1640 medium containing 10% FBS for 6 h before treatment. After preincubation, the cells were transfected with siRNAs or HA‐tagged p62‐expressing plasmid. When studying these cells under conditions of inhibited autophagy, they were treated simultaneously with 1 nmol/L bafilomycin A1 22 or Dimethyl sulfoxide (DMSO) (vehicle control). Three days after these treatments, the cell numbers were evaluated using a Cell Counting Kit‐8 (Dojindo, Kumamoto, Japan).

### Immunofluorescence and transfection of tfLC3 plasmid

HCT8 and HT29 (100,000 cells/coverslip) were plated on glass coverslips (Asahi Techno Glass Corporation, Iwaki Scitech division, Tokyo, Japan) and plasmids expressing tfLC3 and siRNA for p62 were cotransfected into the cells using Lipofectamine 2000 reagent for 48 h. The cells were fixed with 4% paraformaldehyde in phosphate‐buffered saline (PBS) for 15 min and treated with 50 mmol/L NH_4_Cl in PBS for 10 min. Then, the cells were permeabilized with 0.3% Triton X‐100 (Roche Diagnostics) and blocked with 5% normal goat serum albumin (Wako, Osaka, Japan) for 1 h at room temperature. The cells were incubated with mouse monoclonal anti‐p62 antibody (MBL) (1:200 dilution) for 12 h at 4°C. Subsequently, the cells were incubated with Alexa Fluor 405‐conjugated goat anti‐mouse IgG antibodies (Invitrogen) and mounted with ProLong Gold Antifade Reagent (Life Technologies, Inc., Grand Island, NY). These samples were examined under a confocal microscope (C2si, Nikon Instruments, Kawasaki, Japan).

The number of mRFP puncta with or without GFP staining per carcinoma cell was manually counted under the confocal microscope 23 and 20 cells were randomly evaluated in each sample.

### Statistical analysis

In the immunohistochemical studies, associations between the p62 status and clinicopathological factors were evaluated by Student's *t*‐test, cross‐table using the *χ*2 test or Fisher's exact test. Overall survival curves were generated according to the Kaplan–Meier method, and the statistical significance was evaluated using the log‐rank test. Univariate analyses were evaluated by the log‐rank test, and Cox's proportional hazards model was used for the multivariate analysis. Student's *t*‐test was used in the in vitro experiments. JMP^®^ 10 (SAS Institute Inc., Cary, NC) was used for statistical analyses, and differences with *P *<* *0.05 were considered significant.

## Results

### p62 immunolocalization in human colorectal carcinoma

**Figure 1:**
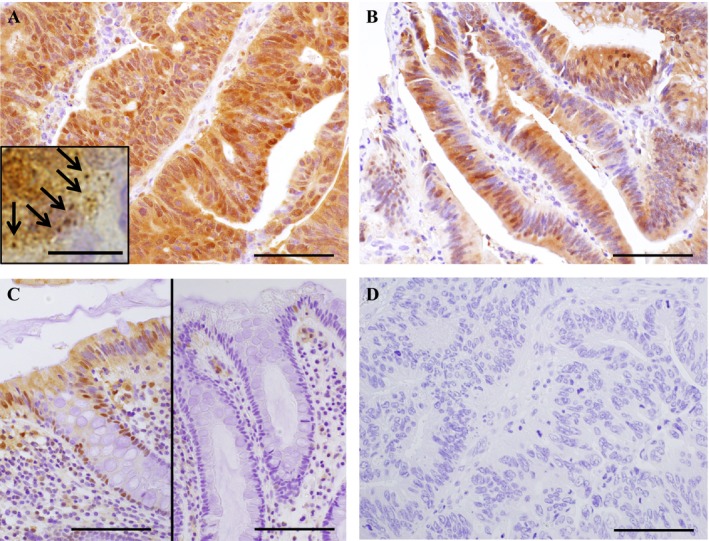
Immunohistochemistry for p62 in colorectal carcinoma. (A) p62 immunoreactivity was detected in the cytoplasm of colorectal adenocarcinoma cells. An inserted photo shows cytoplasmic inclusions in the carcinoma cells at the higher magnification (arrows), suggesting p62 aggregation. (B) p62 immunoreactivity was detected in the cytoplasm of adenoma cells. (C) p62 was weakly and focally immunolocalized in some nonneoplastic epithelial cells (left panel), but a great majority of the nonneoplastic epithelium was negative for p62 (right panel). (D) negative control section of p62 immunohistochemistry (same area as A). Bar = 100 *μ*m, except for an inset (bar = 10 *μ*m).

p62 immunoreactivity was detected in the cytoplasm of the colorectal carcinoma cells (Fig. 1A). Cytoplasmic inclusions were also observed in the carcinoma cells, suggesting p62 aggregates (Fig. 1A, arrows). The status of p62 immunoreactivity was positive in 37 (31%) of 118 colorectal carcinoma cases in our study. p62 immunoreactivity was also detected in the cytoplasm of adenoma cells in 3 (11%) of 28 colorectal adenoma cases (Fig. 1B), but its immunopositivity was significantly lower than that of adenocarcinoma (*P *=* *0.018). p62 was weakly and focally immunolocalized in some nonneoplastic epithelial cells adjacent to the carcinoma (Fig. 1C, left panel), but the p62 status in nonneoplastic epithelium was negative (0%) in all 94 cases examined (right panel) when it was evaluated as adenocarcinoma or adenoma. No significant p62 immunoreactivity was detected in the negative control sections in this study (Fig. 1D).

Associations between the immunohistochemical p62 status and the clinicopathological parameters of the colorectal carcinoma patients are summarized in Table 1. The p62 status was significantly associated with synchronous liver metastasis (*P *=* *0.033), but no significant association was detected between the p62 status and other factors such as patients' age, gender, tumor location, tumor size, histological differentiation, depth of invasion, lymph node metastasis, TNM stage, or Ki‐67 LI in this study.

**Table 1:** Association between p62 immunohistochemical p62 status and clinicopathological parameters in 118 colorectal carcinomas

Value	p62 status		*P* value
	Positive (*n *=* *37)	Negative (*n *=* *81)	
Age (years)^a^	62.6 ± 12.1	64.8 ± 12.6	0.37
Gender			
Men	21	50	0.61
Women	16	31	
Tumor location			
Colon	21	33	0.11
Rectum	16	48	
Tumor size (mm)^a^	52.8 ± 24.3	52.3 ± 18.0	0.89
Histological differentiation			
Tub1 + tub2	36	74	0.43
Por + muc	1	7	
Depth of invasion			
Mucosa–muscularis propria (pT1 + pT2)	7	25	0.17
Through muscularis propria (pT3 + pT4)	30	56	
Lymph node metastasis			
Positive	16	37	0.80
Negative	21	44	
Liver metastasis			
Positive	4	1	**0.033**
Negative	33	80	
TNM stage			
I–II	20	43	0.92
III–IV	17	38	
Ki‐67 LI (%)^a^	50.6 ± 16.2	52.2 ± 18.8	0.47

LI; labeling index.

a The values were presented as mean ± SD, and all other values represented the number of cases. *P* < 0.05 was considered significant, and shown in boldface.

### Correlation between p62 status and clinical outcome of colorectal carcinoma patients

**Figure 2:**
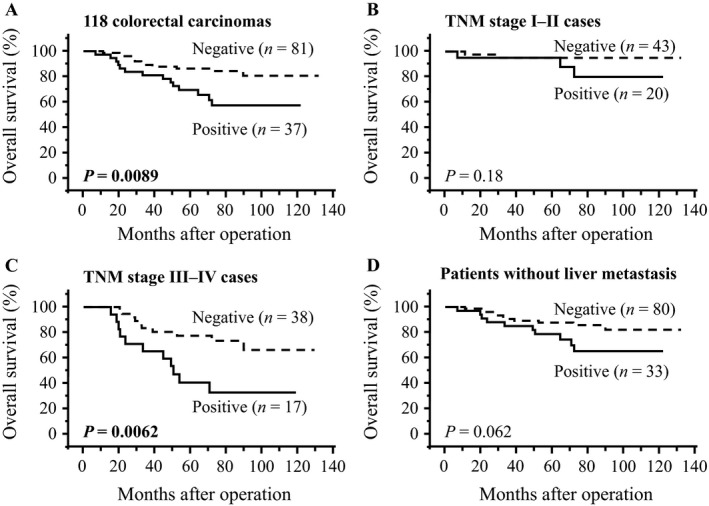
Overall survival curves of colorectal carcinoma patients according to p62 status by Kaplan–Meier method. (A) total cases (*n *=* *118), (B) TNM stage I–II cases (*n *=* *63), (C) TNM stage III‐IV cases (*n *=* *55) and (D) patient group without liver metastasis (*n *=* *113). Solid line summarizes p62‐positive cases and dashed line shows p62‐negative cases. The statistical significance was evaluated using the log‐rank test. *P *< 0.05 was considered significant and is shown in boldface.

As demonstrated in Figure 2A, the p62 status was significantly associated with adverse overall survival in the 118 colorectal carcinoma patients (*P *=* *0.0089). Similar findings were detected regardless of the TNM stage [*P *=* *0.18 in TNM stage I‐II cases (Fig. 2B) and *P *=* *0.0062 in TNM stage III‐IV cases (Fig. 2C)]. The p62 status was positively associated with synchronous liver metastasis in our present study (Table 1), but the p62 status tended to be associated with an adverse prognosis in the patients without synchronous liver metastasis, although the association did not reach statistical significance (*P *=* *0.062) (Fig. 2D).

The results of univariate analysis of overall survival by the log‐rank test (Table 2), synchronous liver metastasis (*P *<* *0.0001), lymph node metastasis (*P *=* *0.0014), p62 status (*P *=* *0.0089), histological differentiation (*P *=* *0.030) and depth of invasion (*P *=* *0.036) were identified as significant prognostic factors for overall survival in these 118 colorectal carcinoma patients. However, the following multivariate analysis demonstrated that only liver metastasis (*P *=* *0.035), lymph node metastasis (*P *=* *0.019), p62 status (*P *=* *0.030), and histological differentiation (*P *=* *0.020) were independent prognostic factors in the patients.

**Table 2:** Univariate and multivariate analyses of overall survival in 118 colorectal cancer patients

Variable	Univariate	Multivariate	
	*P*	*P*	Hazard ratio (95% Cl)
Liver metastasis (+ vs. −)	<**0.0001**	**0.035**	3.97 (1.11–12.57)
Lymph node metastasis (+ vs. −)	**0.0014**	**0.019**	2.98 (1.19–8.14)
p62 status (+ vs. −)	**0.0089**	**0.030**	2.73 (1.10–6.77)
Histological differentiation (mucinous + poor vs. tubular)	**0.030**	**0.020**	4.89 (1.33–14.69)
Depth of invasion (pT3 + pT4 vs. pT1 + pT2)	**0.036**	0.30	1.88 (0.60–8.34)
Tumor location (rectum vs. colon)	0.13		
Tumor size (≥50 vs. <50)	0.63		
Age (years)^a^ ≥64 vs. <64)	0.80		
Ki‐67 LI (%)^a^(≥51.8 vs. <51.8)	0.84		
Gender (women vs. men)	0.84		

*P* < 0.05 was considered significant, and shown as boldface. The significant parameters by univariate analyses were used in the multivariate analysis.

CI; confidence interval, and LI; labeling index.

a The parameters were categorized into two groups according to the median value.

### Effects of p62 expression on cell proliferation in colon carcinoma cells

**Figure 3:**
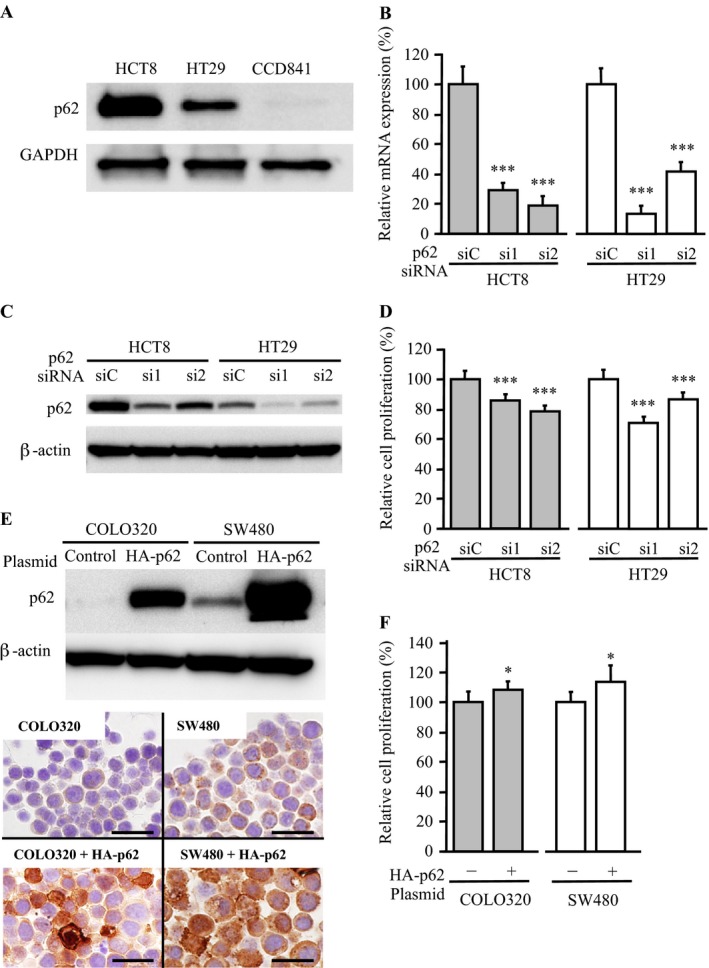
Effects of p62 expression upon cell proliferation in colon carcinoma cells. (A) p62 protein level evaluated by immunoblotting in HCT8, HT29, and CCD841 cells. (B) expression of p62 mRNA evaluated by real‐time PCR in HCT8 (left panel; gray bar) and HT29 (right panel; open bar) cells transfected with p62‐specific siRNA (si1, si2) and negative control siRNA (siC). The values are presented as mean ± SD (*n *=* *3). (C) p62 immunoreactivity in HCT8 and HT29 cells transfected with p62‐specific siRNA by immunoblotting. (D) proliferation assays in the HCT8 and HT29 cells. The values are presented as mean ± SD (*n *=* *6). (E) immunoblotting for p62 in COLO320 and SW480 cells transiently transfected with HA‐tagged p62 plasmid or empty control plasmid (upper panel). Lower panel shows results of immunohistochemistry for p62 in these cell blocks (bar = 20 *μ*m). (F) proliferation assays in the COLO320 and SW480 cells. The values are presented as mean ± SD (*n *=* *6). *P <* *0.05 and ***P <* *0.001 versus control cells (left bar).

Immunohistochemical results suggest that p62 is overexpressed in human colorectal carcinoma cells. When we examined p62 protein levels in human colon carcinoma (HCT8 and HT29) and normal colon epithelial (CCD841) cell lines, p62 protein level was markedly and moderately detected in HCT8 and HT29 cells, respectively, whereas it was negligible in CCD841 cells (Fig. 3A).

Subsequently, we transfected p62‐specific siRNA in HCT8 and HT29 cells to evaluate the biological functions of p62. The p62 mRNA expression levels were significantly (*P *<* *0.001, respectively) decreased in these cells transfected with p62‐specific siRNA (si1 or si2) at 2 days after transfection compared to those transfected with negative control siRNA (siC) (Fig. 3B). The expression level of p62 protein was confirmed by immunoblotting in these cells (Fig. 3C). As shown in Figure 3D, the number of cells was significantly lower in HCT8 cells transfected with p62‐specific siRNAs than in control cells transfected with siC (*P *<* *0.001 for si1 and *P *<* *0.001 for si2) at 2 days after the transfection. Significant association was also detected in HT29 cells transfected with p62‐specific siRNA (*P *<* *0.001 for si1 and *P *<* *0.001 for si2).

To confirm the association between p62 and cell proliferation, we then transiently overexpressed p62 in COLO320 and SW480 colon carcinoma cells. The p62 protein level was negligible and low in COLO320 and SW480 cells, respectively, but it was markedly increased in the cells transfected with HA‐tagged p62 expression plasmid for 2 days (Fig. 3E, upper panel). The accumulation of p62 protein was also confirmed by immunohistochemistry in these cells (Fig. 3E, lower panel). As shown in Figure 3F, the cell proliferative activity was significantly more elevated in COLO320 and SW480 cells transfected with p62 expression plasmid than in control cells transfected with siC at 3 days after the transfection (*P *<* *0.05, respectively).

### Association between p62‐mediated cell proliferation and autophagy in colon carcinoma cells

**Figure 4:**
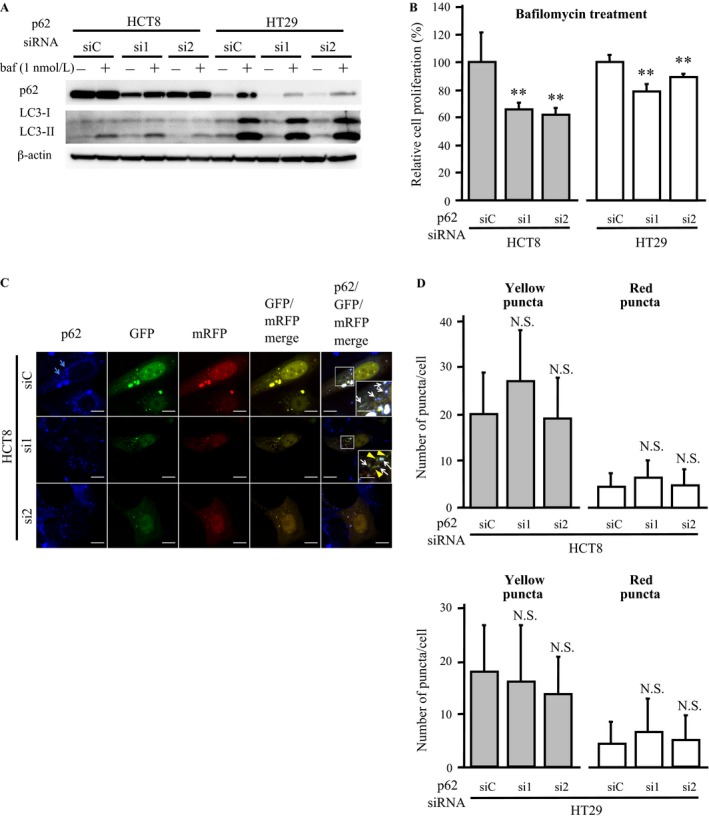
Association between p62‐mediated cell proliferation and autophagy in colon carcinoma cells. (A) immunoblotting for p62 and LC3 in HCT8 and HT29 cells transfected with p62‐specific siRNA (si1, si2) and negative control siRNA (siC). These cells were treated with bafilomycin A1 (1 nmol/L) or vehicle control (DMSO) for 3 days. (B) proliferation assay in the HCT8 and HT29 cells treated with bafilomycin A1. The values are presented as mean ± SD (*n *=* *6). (C) immunofluorescence analysis of HCT8 cells cotransfected with tfLC3 plasmid and p62‐specific siRNA (si1, si2) or control siRNA (siC). p62, GFP, and mRFP staining is shown as blue, green and red, respectively. Blue arrows show puncta formation by p62 staining in the cytoplasm of the carcinoma cells. Colocalization of GFP and mRFP puncta appears as yellow in the GFP/mRFP merged images (i.e., yellow puncta), and colocalization of p62 and yellow puncta appears as white in the p62/GFP/mRFP merged image (bar = 10 *μ*m). In the inset, white arrows indicate yellow puncta with p62 and yellow arrowheads show yellow puncta without p62 (bar = 5 *μ*m). (D) number of yellow and red puncta in HCT8 and HT29 cells cotransfected with tfLC3 plasmid and siRNA for p62. The values are presented as mean ± SD (*n *=* *20). **P <* *0.01 and N.S.; not significant versus control cells (left bar).

p62 is well known to play an important role in selective autophagy 3, 4, 5 and it is therefore very important to examine whether the p62‐mediated cell proliferation is associated with the autophagy activity in colon carcinoma cells. As shown in Figure 4A, treatment with an autophagy inhibitor, bafilomycin A1 (1 nmol/L, 3 days) 22, markedly increased the p62 and LC3‐II (lipidated form of LC3, binding to the autophagosome membrane) protein levels both in HCT8 and HT29 cells transfected with siC and p62‐specific siRNA, demonstrating sufficient suppression of autophagy by bafilomycin A1 treatment 24. However, the cell proliferative activity still remained significantly lower in HCT8 cells transfected with p62‐specific siRNAs than in controls transfected with siC (*P *<* *0.01 for si1 and *P *<* *0.01 for si2) at 3 days after the transfection under the bafilomycin A1 treatment (Fig. 4B, left panel). A similar association was also detected in HT29 cells (*P *<* *0.01 for si1 and *P *<* *0.01 for si2) (Fig. 4B, right panel).

In the immunofluorescence analysis, p62 staining was detected in the cytoplasm with puncta formation in HCT8 cells transfected with siC (Fig. 4C, blue arrows), but it was almost diminished in HCT8 cells transfected with p62‐specific siRNA. We also evaluated the autophagic activity in these cells by the tfLC3 method 21. Briefly, the tfLC3 method is based on the distinction of GFP signal that disappeared due to the lysozomal environment, and the colocalization of GFP and mRFP puncta (i.e., yellow puncta in the merged image) indicates tfLC3 located at the phagophore or autophagosome. In contrast, the mRFP puncta without GFP colocalization (i.e.*,* red puncta) corresponds to an amphisome or autolysosome. Figure 4C demonstrates that yellow puncta were frequently detected in HCT8 cells transfected with siC, and a great majority (~95%) of the yellow puncta was colocalized with p62 in these cells (white arrows). Yellow puncta were also detected in HCT8 cells transfected with p62‐specific siRNA (si1 or si2), but approximately 40% and 70% of these puncta were not colocalized with p62 staining in HCT8 and HT29 cells, respectively (yellow arrowheads). The numbers of yellow and red puncta in HCT8 and HT29 cells transfected with si1 or si2 were similar to those cells transfected with siC (Fig. 4D).

## Discussion

This is the first study to demonstrate that p62 immunoreactivity associated with adverse clinical outcomes in colorectal carcinoma patients. In our present study, p62 immunoreactivity was detected in 31% of the adenocarcinoma cases and 11% of the adenoma cases, but it was almost negligible in the nonneoplastic epithelial cells. Previous studies have reported that p62 immunoreactivity was detected in several human malignancies such as stomach, colon, liver, lung, prostate, breast carcinomas 9, 10, 11, 12, 13, 14, 15, 16, 17, and its immunopositivity ranged from 20% to 95% in these studies. Increased p62 immunoreactivity in colon carcinoma compared to normal epithelium has been reported previously 10, 14, 15, which was consistent with results of our present study. Previously, Takamura et al. showed that multiple benign liver adenomas occurred in autophagy‐deficient mice and additional knockout of p62 lead to the size of the tumors being smaller 25. Therefore, p62 overexpression is suggested to be involved in the progression of colorectal tumors. Our present result also revealed that p62‐immunopositivity was significantly higher in the carcinoma than the adenoma cases, suggesting the particular importance of p62 in colorectal carcinoma.

The results of our in vitro studies demonstrated that p62 significantly promoted the cell proliferation of colorectal carcinoma cells. p62 has an important function as a receptor for selective autophagy 3, 4, 5, and autophagy was reported to be required for the growth of pancreatic carcinoma 26. The results of our present immunofluorescence analyses revealed that p62 was frequently colocalized with yellow puncta (autophagosomes) in the colon carcinoma cells, but the autophagic activity was not linked to the p62 expression level or p62‐mediated cell proliferation in these cells. Previously, Komatsu et al. reported no significant change in the autophagic activity in the liver of p62‐knockout mice 27 and Takamura et al. demonstrated that p62 accumulation caused by a deficiency of autophagy contributed to tumor progression in autophagy‐deficient mice 25. Our present results are in good agreement with these reports above and suggest that autophagy is not directly associated with the p62‐mediated cell proliferation.

p62 also plays an important role as a multifunctional scaffold protein in cell signaling pathways. For instance, p62 overexpression induced by *KRAS* mutation activated NF‐*κ*B signaling pathway and enhanced tumorigenesis in pancreatic ductal adenocarcinoma 6, and NRF2 activation by p62 contributed to tumor growth in hepatocellular carcinoma 7. In addition, Duran et al. reported that p62 promoted tumorigenesis through the activation of the mTORC1 pathway 8. Taken together, these findings and our present results show that p62 is considered to play important roles in the proliferation of colorectal carcinoma, mainly through the activation of cell signaling as a scaffold protein rather than through modulation of the autophagic activity.

Ki‐67 antibody detects cells in all phases of the cell cycle except G0 (resting) phase 28. Ki‐67 LI is frequently used for the assessment of proliferative activity in various tumors, and high Ki‐67 LI in tumor tissues is generally correlated with poor survival in breast and lung carcinomas, astrocytoma, and meningioma patients 29. However, we did not find a significant association between p62 immunoreactivity and Ki‐67 LI in the colorectal carcinoma cases. It is also true that overexpression of Ki‐67 induced growth arrest 30, and Hilska et al. suggested that strongly stained tumor cells had a slow cell cycle and a low proliferation rate 31. In colorectal carcinoma, Valera et al. showed that Ki‐67 is a worse prognostic parameter 32, and Kawakami et al. reported that high Ki‐67 expression was a prognostic factor for liver metastasis 33. However, several investigators also reported that higher Ki‐67 LI was paradoxically associated with longer survival in colon cancer patients 34, 35. Therefore, the significance of Ki‐67 LI still remains controversial in colorectal cancer patients, in contrast to that in other human malignancies.

In this study, p62 immunoreactivity was significantly associated with liver metastasis in colorectal carcinoma. The p62 status was significantly associated with adverse clinical outcomes in the colorectal carcinoma patients, and multivariate analysis revealed that the p62 status was an independent prognostic factor for overall survival in the patients. Recently, Inoue et al. demonstrated p62 as an independent poor prognostic factor for lung cancer‐specific survival 9, and Luo et al. reported that p62 accumulation was an independent prognostic factor for disease‐free survival in triple‐negative breast cancer patients 16. The results of these studies are in good agreement with those in our study. p62 increased migration and invasion in glioblastoma stem cells 36, and p62 immunoreactivity was correlated with distant metastasis in breast carcinoma 17. Moreover, Yu et al. reported that cisplatin‐resistant SKOV3/DDP ovarian carcinoma cells expressed much higher levels of p62 than did cisplatin‐sensitive SKOV3 cells, and knockdown of p62 resensitized SKOV3/DDP cells to cisplatin 37. Therefore, p62 possibly regulates a variety of biological functions in colorectal carcinoma cells in addition to the induction of proliferative activity, and residual carcinoma cells following surgical treatment in p62‐positive colorectal carcinomas could still have a potential for rapid recurrence. We could not examine biological functions of p62 in colorectal carcinoma cells using mouse transplantation model in this study, and further studies are needed to clarify the molecular functions and possible therapeutic potential of p62 in human colorectal carcinoma.

In summary, p62 immunoreactivity was detected in 11% of colorectal adenoma and 31% of the adenocarcinoma cases, while it was almost negligible in the normal epithelium. The p62 status was significantly associated with synchronous liver metastasis in the colorectal carcinoma cases, and it turned out to be an independent adverse prognostic factor for overall survival in the patients. Subsequent in vitro studies revealed that p62 expression levels were significantly associated with the cell proliferation activity of colorectal carcinoma cells, but the p62‐mediated cell proliferation was not correlated with the autophagy activity. These findings suggest that p62 promotes the cell proliferation of colorectal carcinoma mainly as a scaffold protein, and the p62 status in carcinoma cells could serve as a potent prognostic factor in human colorectal cancer patients.

## Conflict of Interest

We declare that there is no conflict of interest associated with this manuscript.
